# Not Ready for Primetime: Challenges of Antenatal Ultrasound in Low- and Middle-Income Country Settings

**DOI:** 10.9745/GHSP-D-17-00213

**Published:** 2017-06-27

## Abstract

Even under optimized trial conditions, antenatal ultrasound was difficult to implement in Equateur Province, DRC. Moreover, the broader study across 5 countries failed to find an impact on pregnancy outcomes. Use of antenatal ultrasound screening appears not to be ready for wide application in low- and middle-income countries.

See related article by Swanson.

There are instances when simple technological fixes can have a major public health impact. For example, investments by the U.S. government and the Bill & Melinda Gates Foundation (and others) to facilitate widespread use of effective vaccines have undoubtedly made an important contribution to reducing the burden of child deaths in low-income countries. But, more commonly in global health, the deployment of an otherwise promising technology is insufficient—on its own—to produce marked improvements in outcomes in the face of real-world complexity. This is well illustrated in an article by Swanson and colleagues,^1^ published in this issue of *Global Health: Science and Practice* (GHSP). The article addresses challenges experienced implementing a field trial. But it is of particular interest to the journal and to many of our readers for lessons that can be drawn on program implementation more broadly.

Swanson and colleagues report on implementation of the First Look Ultrasound study, in which the main intervention consisted of making ultrasound available for routine antenatal screening in peripheral-level health facilities (conducted in 5 countries: Democratic Republic of the Congo [DRC], Guatemala, Kenya, Pakistan, and Zambia). On the face of it, this seems like a straightforward, unequivocal, good thing; routine screening should allow for earlier detection and more timely management—at a suitable level in the health care system—for conditions such as placenta previa, abnormal lie, and twins.

The endpoints of the trial were service utilization (antenatal care, facility birth) and pregnancy outcomes (mortality, morbidity). The article in this issue of GHSP focused on implementation issues encountered, particularly in the most difficult of the study sites, Equateur Province, DRC. From this experience, the authors make the point that *threshold conditions* need to be met for feasibility; in the DRC site, such conditions were stretched to the limit. Specific challenges encountered were:

- Power supply (special arrangements needed to be made to install solar panels)
- Equipment maintenance and repair (costs, delays when repairs were needed)
- Supply chain for consumables
- Security, as the ultrasound equipment was expensive and therefore an attractive target for theft—this required complicated logistical arrangements
- Availability of clinical staff—the study hired its own nurses to do the screening, due to logistical challenges transporting equipment and concern about protocol adherence by regular nursing staff (including documentation) in the absence of close supervision
- Functional referral to a center capable of providing comprehensive emergency obstetrical care (including blood, anesthesia) and geographically and financially accessible to potential users
- Streamlining/coordination to reduce procedural barriers for patients at the receiving health facility
- Quality assurance for ultrasound diagnostics

If implementation under comparatively well-resourced trial conditions turned out to be very challenging, how much more so would it be under routine conditions?

Although not the focus of the article published in GHSP, the authors have published overall results of their trial elsewhere.^2^ With pregnancy outcomes as their main endpoint, their multicountry trial failed to find an impact.

An intervention or a technology may have high face validity. That is to say, it may seem like a no-brainer that it should be deployed and that, having done so, one should expect it to produce a benefit. But generally speaking, interventions or technologies are embedded in systems with many other moving parts.

For routine obstetrical ultrasound screening, even in high-income settings it is unclear how much net benefit this yields.^3^^,^^4^ Based on the results of the First Look study,^2^ conducted in low-income country settings, the investigators succeeded—with considerable effort—in delivering the screening intervention; across the whole study 78% got at least 1 ultrasound and, of those for whom screening detected a high-risk condition, 71% completed referral. However, there were no clear benefits with regard to either increased use of antenatal care or hospital births or improved birth outcomes. In their publication of the main effects of the trial, the authors rightly conclude that “introducing routine [obstetrical] ultrasound screening in low and middle income countries is unlikely to improve outcomes and would potentially pose a large burden on available resources, and detract from other more beneficial services.”^2^

In the first instance, an intervention needs to be efficacious. That is to say that it should produce net benefit, at least under optimal conditions. But secondly, it must be feasible to deliver it, without detracting from other services. It is clear that this intervention, in this kind of setting, is not ready for prime time on either count. –*Global Health: Science and Practice*
